# Fat-Phobic and Non-Fat-Phobic Anorexia Nervosa: A Conjoint Analysis on the Importance of Shape and Weight

**DOI:** 10.3389/fpsyg.2020.00090

**Published:** 2020-01-31

**Authors:** Julia Korn, Silja Vocks, Lisa H. Rollins, Jennifer J. Thomas, Andrea S. Hartmann

**Affiliations:** ^1^Department of Psychiatry and Psychology, Universität zu Lübeck, Lübeck, Germany; ^2^Department of Psychology, University of Osnabrück, Osnabrück, Germany; ^3^Massachusetts General Hospital, Harvard Medical School, Boston, MA, United States

**Keywords:** non-fat phobic anorexia nervosa, importance of shape and weight, conjoint analysis (CA), indirect measure, fear of weight gain

## Abstract

With the introduction of new diagnostic criteria in DSM-5, fear of weight gain no longer represents a sine qua non-criterion for the diagnosis of anorexia nervosa (AN). This is of relevance as a subgroup of individuals with AN denies fear of weight gain as the reason for restrictive eating but still remain at a very low weight. As self-reports are susceptible to bias, other methods are needed to confirm the existence of the subtype in order to provide adapted treatment. Therefore, we aimed to measure fear of weight gain using a novel method in clinical psychology, the conjoint analysis (CA). Relative importance and preference scores for various life aspects, including appearance/shape and weight were assessed in women with fat-phobic AN (FP-AN, *n* = 30), NFP-AN (*n* = 7), and healthy controls (*n* = 29). Individuals with FP-AN showed a significant lower preference for weight gain versus weight maintenance than HC (*p* = 0.011, $\eta_{\text{p}}^{2}$ = 0.107). Correlation between explicitly assessed drive for thinness and CA score was low. As expected, in FP-AN the explicitly endorsed fear of weight gain was confirmed by the marked preference for weight maintenance compared to HC, while for NFP-AN explicit and implicit measures diverged, indicating that against their self-report they may experience at least some fear of weight gain. The utility of CA as a tool to measure fear of weight gain — and potentially other psychopathological constructs —requires further confirmation.

## Introduction

Individuals with anorexia nervosa (AN) who do not report fear of gaining weight or becoming fat, so called non-fat phobic anorexia nervosa (NFP-AN; Lee et al., 1993), seem to occur with a wide geographic distribution in both western and non-western populations and exhibit a consistent profile of low scores on measures of eating disorder pathology (Dalle Grave et al., 2008; Becker et al., 2009; Wildes et al., 2013). The changes made to the diagnostic criteria for AN within the fifth edition of the *DSM* (*DSM-5*, American Psychiatric Association [APA], 2013) which saw Criterion B altered to no longer make fear of weight gain a prerequisite for the diagnosis of AN, allow clinicians to classify NFP-AN as a *bona fide* variant of AN. Furthermore, while individuals with NFP-AN might exhibit lower psychopathology (Forbush and Wildes, 2017), they also present with higher treatment drop-outs, lower remission rates, and lower insight into their condition (Santonastaso et al., 2009). The latter could be one possible explanation for their denial of fear of weight gain, besides having divergent rationales for food restriction (Becker et al., 2009; Lee et al., 2012) and minimizing or denying shape and weight concerns (Izquierdo et al., 2019). Thus, an identification and diagnosis of individuals with NFP-AN are essential to adequately shape treatment for this patient group, potentially with a less strong focus on alteration of body image disturbance.

An additional reason for the challenging nature of determining how NFP-AN should be classified is the manner in which the construct has been measured in the past. The research referenced above assessed the existence of fear of weight gain through self-report using explicit measures such as questionnaires, e.g., the Eating Disorder Inventory (EDI) (Garner et al., 1983), and especially its subscale Drive for Thinness, being vulnerable to underreporting due to denial of illness in AN (Vandereycken, 2006). However, there have been calls for the use of performance-based measures in NFP-AN to determine whether fear of weight gain may be present on an implicit level even if not explicitly endorsed (Thomas et al., 2013). The only study aiming to implicitly assess fear of weight gain in AN made use of implicit association tests (IATs) (Izquierdo et al., 2019). Results revealed that adolescents with and without reported fear of weight gain showed similar implicit biases toward dieting and thinness, but those with NFP-AN scored significantly lower on the explicit EDI-3 Drive for Thinness subscale than individuals with FP-AN. The lack of group differences on implicit measures may be ascribed to the use of a categorical approach to assess fear of weight gain. Yet, a dimensional approach to examine the association of implicit and explicit measures of fear of weight gain and eating psychopathology is lacking. This study highlights the novel information that can be gained by investigating both implicit and explicit measures in this group.

In addition to the IAT, a Conjoint Analysis (CA) could be a novel implicit measure to assess the relative importance of shape and weight. CA is a well-established method that has been predominantly used in market research and allows for the analysis of individual preferences for products in order to infer the relative importance of the features making up these products (Wittink and Cattin, 1989; Wittink et al., 1994). CA has been shown to be a reliable method to assess the view of a person (Ryan et al., 2001). Even though CA has mainly been utilized in commercial settings, it has recently gained popularity in psychological and medical settings and has been shown to be useful in evaluating wishes and attitudes in clinical populations (Haarig and Mühlig, 2015). In a related context, CA was used to detect implicit weight-based discrimination that participants denied when questioned explicitly (Caruso et al., 2009). It has, however, not been used to assess clinical symptoms before. There is a particular type of CA that has gained much popularity in recent years, i.e., the choice-based conjoint (CBC) analysis (Gensler, 2006), in which product preferences are measured indirectly by observing an individual’s choices between alternatives. For example, respondents may be presented with and asked to choose between different mobile phone product profiles, which vary on characteristics such as brand, size, resolution, and price. Of each characteristic at least two variations should exist, e.g., at least two sizes of mobile phones, two prices, two brands and resolutions. These variations of characteristics were termed attributes for this piece of research. From the choices participants make, the relative importance score for each characteristic as well as the preference for the attributes within this characteristic (preference scores) are derived.

AN has an overpowering impact on all aspects of a patient’s life, affecting relationships with friends, family and partners, the ability to perform within school or work settings and engage in pleasure activities (De Ruysscher et al., 2015). Thus, an examination of the relative importance of weight compared to these other factors in life may shed light on the discussion on denial of fear of weight gain in some individuals with AN. Therefore, we created life profiles representing scenarios that participants could choose for their own lives based on factors pertaining to interpersonal relationships, success, hobbies, security, and appearance/shape and weight, with the latter being the factor used as an indicator of fear of weight gain. These life profiles contained attributes that represented two opposing manifestations of these factors (e.g., factor: Appearance/Shape and Weight, attributes: weight maintenance and weight gain). Fifteen choice sets including two life profiles each were presented to the participants who had to choose one profile per choice set. It is important to note, that participants had to make a relative choice, e.g., between maintaining weight and not having a partner (see Table 1, profile 1) versus gaining weight and being in a stable partnership (profile 2).

**TABLE 1:** Example of a choice set presented within the choice based conjoint analysis.

**Choice Set 1**	

**Profile 1**	**Profile 2**
Don’t have a partner	Are in a stable partnership
Have good relationships with friends and family	Don’t have rewarding relationships with friends and family
Don’t have any hobbies that give you joy	Don’t have any hobbies that give you joy
Maintained your weight in the last 2 months and fit into your favorite jeans	Gained 3 kg in the last 2 months and don’t fit into your favorite jeans
Are successful at work or school	Are successful at work or school
Worry about your financial situation	Worry about your financial situation

The aim of this study was to test a novel implicit measure, that may be a useful tool for future discriminative analysis of absence of fear of weight gain in a subgroup of participants with AN. As a first step, we compared fear of weight gain in participants with FP-AN and healthy controls (HC) through an implicit or indirect measure, more specifically by measuring the importance of the factor “Appearance/Shape and Weight” and preference for attributes related to fear of weight gain when making choices between life profiles. Furthermore, for a dimensional approach to examine fear of weight gain, we aimed to investigate whether the extent of implicitly assessed importance and preference scores is associated with the extent of explicit questionnaire-based measures of eating disorder pathology, specifically fear of weight gain and body image disturbance in participants with FP-AN and NFP-AN. Additionally, we aimed to conduct a preliminary exploratory analysis on the ratings of the CBC in a small subgroup of participants with NFP-AN.

We hypothesized that (1) participants with FP-AN would show significantly higher importance ratings on the factor “Appearance/Shape and Weight” than HC. Furthermore, we hypothesized that (2) participants with FP-AN would show a significantly higher preference for the attribute pertaining to weight maintenance versus weight gain within the factor “Appearance/Shape and Weight” than HC. We also hypothesized that (3) the relative importance score for the factor “Appearance/Shape and Weight” would show a significant positive, and the preference for the attribute pertaining to weight gain would show a significant negative correlation with explicit questionnaire-based measures of fear of weight gain and body-image disturbance.

## Materials and Methods

### Participants

HC and participants with FP-AN and NFP-AN were recruited via clinics, psychotherapy out-patient treatment centers, counseling services, Facebook and other online communities, advertisements on online marketspaces such as eBay, digital noticeboards and noticeboards at universities, university mailing lists, and advertisements in local newspapers. Prospective participants contacted the research team via e-mail and an appointment for an initial telephone screening was agreed upon. During the telephone screening, the prospective participants were assessed for the following inclusion criteria: Being female; a minimum age of 15 years; fluent in written and spoken German as well as no history of mania, psychosis, substance abuse or suicidal ideation. Due to the nature of the experiments carried out within a larger project this study was part of, participants could have no pre-existing eye conditions or neurological disorders, and no history of epilepsy. HC could not have experienced any lifetime mental health disorder. Prospective participants with AN had to answer at least two of the five items of the SCOFF questionnaire (Morgan et al., 2000) with *yes* and have a body mass index (BMI) below 18.5 kg/m^2^ in order to be invited to a further diagnostic appointment at the laboratory. From a total of *n* = 118 individuals who took part in the telephone screening, *n* = 51 had to be excluded as they met exclusion criteria. Participants with AN were classified as FP- or NFP-AN by means of the Drive for Thinness scale of the EDI-2 (Paul and Thiel, 2004; see below), with participants receiving a score equal to or below 7 being classified as NFP-AN and participants receiving a score above 7 being classified as FP-AN, which is in line with the classification in previous research (Becker et al., 2009).

### Procedure

After arrival at the laboratory of the university outpatient clinic, participants first completed the informed consent process and additional parental assent was obtained for participants below the age of 18. The appointment typically lasted for 4–5 h. The lead researcher, a certified clinical psychologist, then completed structured clinical interviews (see below) with them, which would take up to 60 min. Unless the interviews indicated that inclusion criteria were not met (*n* = 1), research assistants completed the remaining tasks with the final set of participants (*N* = 66, HC: *n* = 29, FP-AN: *n* = 30, NFP-AN: *n* = 7). First, each participant was measured and weighed. Subsequently, a headless photo of the participants in standardized underwear was taken that was used for paradigms conducted after the CBC (eye-tracking, Hartmann et al., submitted; electroencephalography and startle, unpublished data). Afterward, participants were led to another room containing a laptop for the completion of the CBC. Participant were familiarized with the laptop and given instructions for the CBC. Participants were left alone for the completion of the CBC and given as much time as required. After the CBC, participants completed a subsequent Implicit Association Task (unpublished data) as well as the above mentioned paradigms. They also completed the questionnaires detailed below. Finally, the participants attended a debriefing with the lead researcher in which it was ensured that they had not suffered any major distress and were informed of the exact aims of the study, as well as paid the incentive of 70€. This study received ethical approval by the university’s ethics committee of the last author.

### Conjoint Analysis Task

As a basis for our CBC, we determined the following six factors as relevant: “Belonging/Intimacy,” “Belonging/Interpersonal,” “Leisure/Hobbies,” “Appearance/Shape and Weight,” “Success in Job or School,” and “Security/Finances.” Each factor contained two attributes which represented opposing manifestations of this factor. Of particular importance was the factor “Appearance/Shape and Weight” through which we assessed fear of weight gain. The attributes within this factor are very close in wording to items from the Anorexia-Fear-Scale (Schulze and Keller, 2009), a questionnaire developed to fear of weight gain which has been demonstrated to be a good indicator of this construct. The other factors were chosen in expert consensus since they represent aspects through which people tend to define their lives and are often heavily impacted by AN (De Ruysscher et al., 2015).

Upon entry of these factors and attributes into XLSTAT (XLSTAT 2014, 2014, Paris, France), so-called life profiles were created automatically using a full-profile, fractional factorial design. In a full-profile design participants are asked to make choices between complete profiles containing an attribute from each factor, as opposed to making choices between single attributes from within the same factor. This design was chosen as this is more closely modeled on choice scenarios which people might experience in real life and thus has higher external validity (Backhaus et al., 2016). The use of a fractional factorial design, i.e., a suitable fraction of all possible combinations of the factors, was necessary as a full factorial design would have resulted in a number of stimuli exceeding the amount participants can reliably evaluate (Backhaus et al., 2016). The typical CA design consisted of a median of 16 choice sets (Wittink and Cattin, 1989). Choice sets were generated applying an incomplete block design, i.e., each choice set contained only a selection of attributes of each factor, thus not all attributes appear in all of the choice sets (i.e., single attributes might be the same between the two profiles of each choice sets). A total of 15 choice sets containing two life profiles each were created and presented within an excel sheet. Table 1 displays an example of a typical choice set used in our CBC, in which the two profiles share some attributes and also differ in others.

As participants do not rate the attributes themselves but a set of profiles formed by a combination of attributes at different levels, problems of social desirability are mitigated (e.g., Wallander, 2009; Horiuchi et al., 2018). Moreover, CA was shown to be more resistant to socially desirable responses than any other commonly used self-report measures, e.g., Likert-type choices (Tomassetti et al., 2016). Hence, also CBC designs are useful when sensitive features are assessed and the need for cover stories to mask the goal of the experiment is low (Dahl, 2018). In line with this, in our study, we did not use a cover story for the CBC, but rather told the participants to accomplish a choice task in which they select their preferred scenario from different fictive scenarios. Participants were instructed to read the given profiles carefully and then choose the profile that was most in line with what they would wish for in their own lives. Then, they were asked to enter their chosen profile into a box next to each choice set and scroll down to view the next two profiles. The utility-theoretic approach of discrete choice models postulates that respondents will choose the alternative that provides the highest possible utility to them (Eisen-Hecht et al., 2004). It is assumed that the utility of a product is determined by the individual, so called part-worth utilities assigned by the respondent to the attributes contained within it (Backhaus et al., 2016). The attributes are defined as the various manifestations or levels within a factor. The part-worth utilities thus describe the preference of a respondent for a specific attribute relative to the other attributes within the same factor. For this piece of research, it was assumed that part-worth utilities assigned to attributes by HC and participants with FP- and NFP-AN would be significantly different.

### Measures

The following instruments were administered to participants in the following order:

*Structured Clinical Interview for DSM-IV* (SKID; German-language version: Wittchen et al., 1997): The SCID I is a semi-structured diagnostic interview used to determine Axis I disorders. For this study, the SCID I was used to determine if exclusion criteria were met. The interrater reliability is satisfactory to good (0.61 ≤ *r*_*icc*_ ≤ 0.83; Lobbestael et al., 2011).

*Eating Disorder Examination* (EDE 12.0D; German-language version: Hilbert and Tuschen-Caffier, 2006): The EDE interview assesses eating-disorder psychopathology and consists of a total of 22 items which can be assigned to the following four subscales: Restraint, Eating Concern, Weight Concern and Shape Concern. Another six items that are not included in the total or subscale scores are used for diagnostic purposes. For the present study, we made use of only three items, namely Importance of Shape, Importance of Weight, and Fear of Weight Gain, summarized to one sum score. The internal consistency of this EDE score in this study was good (Cronbach’s α = 0.87).

*Eating Disorder Inventory 2* (EDI-2; German-language version: Paul and Thiel, 2004): The EDI-2 consists of 91 items measuring eating-disorder psychopathology. In this study we used only the subscale Drive for Thinness subscale, which assesses the frequency of behaviors, thoughts and feelings that concern drive for thinness and fear of weight gain. The EDI-2 subscale Drive for Thinness showed acceptable internal consistency in the current study (Cronbach’s α ≤ 0.77).

*Body Checking Questionnaire* (BCQ; German-language version: Vocks et al., 2008): The BCQ consists of 23 items and measures body checking behavior related to eating disorders associated with a negative body image. The total score had excellent internal consistency in our sample (Cronbach’s α ≤ 0.95).

*Body Image Avoidance Questionnaire* (BIAQ; German-language version: Legenbauer et al., 2007): The BIAQ is a questionnaire made up of 19 items measuring avoidance behaviors regarding clothing and social activities. The total score had good internal consistency in the current study (Cronbach’s α = 0.89).

### Statistical Analysis

To evaluate demographic and psychosocial differences between groups, we conducted univariate analyses of variance (ANOVAs) with Bonferroni-corrected *post hoc* comparisons (for continuous variables) and Kruskal–Wallis H test with *post hoc* pairwise Dunn–Bonferroni comparisons or χ^2^ tests (for categorical variables). The further statistical analyses consisted of two main stages: The analysis of group differences in the CBC analysis and the correlational analysis of the CBC results and questionnaire data.

#### Group Differences in the CBC Analysis

The Hierarchical Bayes analysis (Allenby and Rossi, 2006), conducted in XLSTAT, follows an iterative process in which empirical choice data is used to estimate individual part-worth utilities, which in turn are used to estimate the distribution of these values on an aggregate level. The aggregate level data is then used to improve the part-worth utility estimates. This process is repeated until estimates cannot be improved further. Based on the individual and aggregate level part-worth utilities the importance of factors within the decision-making process can be determined. For this the ranges of part-worth utilities within each factor are calculated and converted into percentages, resulting in relative importance scores summing up to 100%. The part-worth utilities estimated by XLSTAT were converted to zero-centered differences for the purpose of further analysis. This was necessary in order to counteract the potential effects of response error within the sample on part-worth utilities and to ensure accurate results of the significance tests which were conducted thereafter.

We conducted all subsequent analyses in SPSS version 24.0 (IBM Corp., 2016, Chicago, IL, United States). The relative importance scores for the factors “Belonging/Intimacy,” “Leisure/Hobbies,” “Appearance/Shape and Weight,” and “Success in Job or School” showed significant skew and kurtosis. Log and square root transformations failed to produce non-significant skew and kurtosis, so we used the non-parametric Mann–Whitney *U* test to test for group differences within these factors. We tested the remaining factors and attributes for significant differences between groups with independent one-way analyses of variance (ANOVA). Pairwise comparisons were conducted via Bonferroni tests as this is recommended for use if the number of contrasts of interest does not exceed the number of factor levels (Kao and Green, 2008). As measures of effect size, partial η^2^ for parametric and η^2^ for non-parametric tests were reported (small = 0.01; medium = 0.06; large = 0.14).

#### Correlational Analysis

We calculated a total of 6 correlations across all participants with AN. The BIAQ, BCQ, EDI-2 Drive for Thinness, and sum score of the items Importance of Weight, Importance of Shape and Fear of weight gain from the EDE were each correlated with the relative importance score for the factor “Appearance/Shape and Weight” and the part-worth utility of the attribute “Gained 3 kg in the last 2 months and don’t fit into your favorite jeans.” The part-worth utility for the aforementioned attribute and the scores from all of the above questionnaires showed significant skew and kurtosis. Log and square root transformations failed to produce non-significant skew and kurtosis for all of the above except the scores obtained from the BCQ for which a log transformation was performed. For this reason, we calculated the non-parametric Spearman correlation for all correlations mentioned above apart from the correlation between the BCQ score and the relative importance score for which we calculated a Pearson’s correlation (*r* small = 0.1; medium = 0.3; large = 0.5).

#### Exploratory Analysis of Subgroup Differences

The relative importance scores for the factors “Belonging/Intimacy,” “Belonging/Interpersonal,” “Success in Job or School,” and “Finance” showed significant skew and kurtosis, so we used the non-parametric Mann–Whitney *U* test to test for group differences within these factors. We tested the remaining factors and all attributes for significant differences between groups with independent one-way analyses of variance (ANOVA).

## Results

### Participants’ Characteristics

Table 2 presents sociodemographic and clinical characteristics of the three groups. Groups did not significantly differ in age, but as expected both groups with AN had a significantly lower BMI than the HC group. In all questionnaire scores, FP-AN scored significantly higher than HC and, except from the sum score of the EDE items, also significantly higher than NFP-AN.

**TABLE 2:** Sample characteristics.

	**HC (*n* = 29)**	**NFP-AN (*n* = 7)**	**FP-AN (*n* = 30)**	**Test statistic (*F or H*)**	**Test χ^2^ (df = 2)**
Age (*M*, *SD*, range) years	23.28 (2.52, 19–28)	27.14 (11.67, 15–45)	25.53 (10.43, 17–58)	0.49	
BMI (*M*, *SD*)	21.41(2.19)^b,c^	14.54(3.80)^a^	16.14(2.65)^a^	37.28***	
Marital status, *n* (%)					
In a romantic relationship	21 (72.41)	3 (42.86)	5 (6.67)		18.61***
Drive for Thinness EDI-2 (*M*, *SD*)	0.90(2.53)^c^	2.71(2.87)^c^	13.57(4.12)^a,b^	50.61***	
BCQ total score (*M*, *SD*)	39.48(11.19)^c^	43.57(17.83)^c^	62.57(18.80)^a,b^	16.54***	
BIAQ total score (*M*, *SD*)	37.76(7.13)^c^	42.14(8.93)^c^	58.87(9.04)^a,b^	39.17***	
Sum score EDE items Importance of Weight, Importance of Shape and Fear of Weight Gain (*M, SD*)	2.28(2.22)^c^	7.57 (5.94)	12.47(4.34)^a^	41.34***	
Current psychotherapy, *n* (%)		4 (57.14)	7 (23.33)		12.42**
Past psychotherapy, *n* (%)					
Residential		5 (71.43)	15 (50.00)		27.66***
Semi-residential/day clinic		0 (0.00)	2 (6.66)		2.48
Out-patient		6 (85.72)	15 (50.00)		27.48***

Superscripts denote significant differences from the two other groups: a = HC, b = NFP-AN, c = FP-AN. **p < 0.01, ***p < 0.001.

### Group Differences in the CBC Analysis

Table 3 displays the estimation results of the CBC analysis by group. The report of results of the CBC analysis are started with the presentation of the factor “Appearance/Shape and Weight” as this is the one in focus in this manuscript, followed by the other factors in order of display in Table 3.

**TABLE 3:** Part-worth utilities and relative importance in the choice based conjoint analysis by group.

**Factors**	**Attributes**	**HC**	**FP-AN**	**HC**	**FP-AN**
Belonging/Intimacy	Are in a stable partnership	−12.5(26.71)*	6.5(33.50)*	8%(6.00)	9%(6.85)
	Don’t have a partner	12.5(26.71)*	−6.5(33.50)*		
Belonging/Interpersonal	Have good relationships with friends and family	109.0 (39.57)	99.8 (49.50)	37%(10.74)	35%(12.22)
	Don’t have rewarding relationships with friends and family	−109.0(39.57)	−99.8(49.50)		
Leisure/Hobbies	Have rewarding hobbies	−26.2(50.29)	−2.9(49.73)	16%(9.36)	13%(9.78)
	Don’t have any hobbies that give you joy	26.2 (50.29)	2.9 (49.73)		
Appearance/Shape and Weight	Maintained your weight in the last 2 months and fit into your favorite jeans	−16.6(34.40)*	6.6(33.78)*	11%(5.98)	9%(6.22)
	Gained 3 kg in the last 2 months and don’t fit into your favorite jeans	16.6(34.40)*	−6.6(33.78)*		
Success in Job or School	Are successful at work or school	32.5(38.78)*	−10.4(50.98)*	13%(10.08)	15%(8.96)
	Have problems at work or school	−32.5(38.78)*	10.4(50.98)*		
Security/Finances	Feel financially secure	3.6 (55.67)	−1.2(63.85)	14%(11.38)	19%(9.49)
	Worry about your financial situation	−3.6(55.67)	1.2 (63.85)		

*p < 0.05 for Bonferroni test.

There were no significant differences between the relative importance scores of the groups for the factor “Appearance/Shape and Weight,” *U* = 353.00, *p* = 0.214, η^2^ = 0.026. For both groups, HC and participants with FP-AN, it was the fifth most important. A significant effect for group on part-worth utilities was found, *F*(1,57) = 6.84, *p* = 0.011, $\eta_{\text{p}}^{2}$ = 0.107, with participants with FP-AN showing a significantly higher preference for the attribute pertaining to maintaining weight versus gaining weight than HC.

The factor “Belonging/Intimacy” held the lowest relative importance when choosing a life profile for all groups. The part-worth utilities of the attributes within this factor reveal that both groups of participants with FP-AN preferred being in a stable relationship over not having a partner, whilst HC gave preference to the latter in order to choose positive attributes in other life domains. Relative importance scores, *U* = 473.00, *p* = 0.565, η^2^ = 0.006 did not vary significantly across groups. The part-worth utilities revealed a significant higher preference for being in a partnership for participants with FP-AN than HC, *F*(1,57) = 3.04, *p* = 0.019, $\eta_{\text{p}}^{2}$ = 0.093.

The factor “Belonging/Interpersonal” had the greatest impact on the choice of life profile for HC and participants with FP-AN. The relative importance ratings for this factor did not vary significantly across groups, *F*(1,57) = 0.49, *p* = 0.485, $\eta_{\text{p}}^{2}$ = 0.009. The part-worth utilities of the attributes within this factor reveal a pronounced preference for rewarding relationships with friends or family over a lack of these for all groups. Part-worth utilities did not vary significantly across groups for these attributes, *F*(1,57) = 0.62, *p* = 0.433, $\eta_{\text{p}}^{2}$ = 0.011.

“Leisure/Hobbies” was the second most important factor when choosing a life profile for HC, the fourth most important for participants with FP-AN. No significant effect for group on relative importance scores for this factor was detected, *U* = 341.00, *p* = 0.154, η^2^ = 0.034. The part-worth utilities showed that all groups had a stronger preference for not having any rewarding hobbies over having rewarding hobbies in order to get what they want in other life domains, with no significant differences between groups, *F*(1,57) = 3.20, *p* = 0.079, $\eta_{\text{p}}^{2}$ = 0.053.

No significant effect was found on the relative importance scores for the factor “Success in Job or School,” *U* = 488.00, *p* = 0.422, $\eta_{\text{p}}^{2}$ = 0.011. Participants with FP-AN rated it the third most important factor and for HC the fourth most important. A significant effect for group on the part-worth utilities was also shown, *F*(1,57) = 13.16, *p* = 0.001, $\eta_{\text{p}}^{2}$ = 0.188, with HC showing a significantly higher preference for being successful at work or school than participants with FP-AN.

The factor “Security/Finances” was second most important for participants with FP-AN and third most important for HC and participants with NFP-AN, with no significant effect for group on relative importance of this factor, *F*(2,63) = 1.42, *p* = 0.250, $\eta_{\text{p}}^{2}$ = 0.043. No significant effect for group on part-worth utilities could be detected either *F*(1,57) = 0.10, *p* = 0.759, $\eta_{\text{p}}^{2}$ = 0.002. HC also showed preference for feeling financially secure. Participants with FP-AN showed a preference for worrying about their financial situation.

### Correlational Analyses

We found no significant correlations between the part-worth utility, the relative importance score of the factor “Appearance/Shape and Weight,” and questionnaires. Table 4 displays the results of the correlational analyses.

**TABLE 4:** Correlations between relative importance of Appearance and part-worth utility of Weight Gain in the choice based conjoint analysis results and questionnaires.

	**Drive for**	**BCQ**	**BIAQ**	**Sum score**
	** thinness**	**total**	**total**	** of three**
	****(EDI-2)****	** score**	**score**	**EDE-items^1^**
**Relative Importance**				
Appearance/Shape and Weight	−0.06	−0.16	−0.16	−0.31
**Part-Worth Utility**				
Gained 3 kg in the last 2 months and don’t fit into your favorite jeans	−0.04	−0.14	−0.02	−0.14

*^1^Items: Importance of Weight, Importance of Shape, Fear of Weight Gain.*

### Exploratory Analyses of Subgroups

No significant effect for group was found on the relative importance of the factor “Appearance/Shape and Weight,” *F*(1,34) = 1.74, *p* = 0.179, $\eta_{\text{p}}^{2}$ = 0.049, being the fourth most important for participants with NFP-AN. The part-worth utilities did not vary significantly across both groups, *F*(1,34) = 2.66, *p* = 0.112, $\eta_{\text{p}}^{2}$ = 0.072, but there was a pronounced preference for weight maintenance over weight gain in participants with NFP-AN, part-worth-utility of 9.6 (*SD* 52.72) vs. −16.6 (*SD* 33.78) for HC.

There were no significant differences between the relative importance scores of the groups for the factor “Belonging/Intimacy,” *U* = 100.00, *p* = 0.969, η^2^ = 0. No significant effect for group on part-worth utilities could be detected either, *F*(1,34) = 1.37, *p* = 0.249, $\eta_{\text{p}}^{2}$ = 0.039. Participants with NFP-AN preferred being in a stable relationship over not having a partner, whilst HC gave preference to the latter in order to choose positive attributes in other life domains.

The factor “Belonging/Interpersonal” was the second most important for those with NFP-AN. The relative importance ratings for this factor varied significantly across groups, *U* = 39.0, *p* = 0.011, η^2^ = 0.173. Part-worth utilities also varied significantly across both groups for these attributes, *F*(1,34) = 6.49, *p* = 0.016, $\eta_{\text{p}}^{2}$ = 0.160, with participants with NFP-AN showing significantly lower preference for having rewarding relationships with friends and family than HC.

“Leisure/Hobbies” was the fifth most important factor for participants with NFP-AN. No significant effect for group on relative importance scores for this factor was detected, *F*(1,34) = 1.38, *p* = 0.248, $\eta_{\text{p}}^{2}$ = 0.039. The part-worth utilities revealed no significant differences between groups, *F*(1,34) = 0.62, *p* = 0.436, $\eta_{\text{p}}^{2}$ = 0.018.

For participants with NFP-AN, the factor “Success in Job or School” was the most important when choosing a life profile, placing significantly more importance on it than HC (*U* = 151.00, *p* = 0.049, η^2^ = 0.109). No significant effect for group on part-worth utilities was found, *F*(1,34) = 0.70, *p* = 0.408, $\eta_{\text{p}}^{2}$ = 0.020.

The factor “Security/Finances” was third most important for both groups, with no significant effect for group on relative importance of this factor, *U* = 121, *p* = 0.456, η^2^ = 0.017. No significant effect for group on part-worth utilities could be detected either *F*(1,34) = 0.75, *p* = 0.394, $\eta_{\text{p}}^{2}$ = 0.021.

## Discussion

The aim of this study was to test a novel implicit measure of fear of weight gain in participants with AN and HCs, specifically by measuring the relative importance of appearance and preference for attributes related to fear of weight gain when making choices between various life profiles with the help of a CBC analysis. Furthermore, for a dimensional approach to fear of weight gain we aimed to examine the correlations between implicit and several explicit measures of fear of weight gain and behavioral manifestations of a body-image disturbance.

In disagreement with our first hypothesis, individuals with FP-AN and HC did not differ on the relative importance of the factor “Appearance/Shape and Weight.” Our second hypothesis was confirmed since participants with FP-AN showed a significantly lower preference for weight gain versus weight maintenance compared to HC. Contrary to the third hypothesis, all correlations between implicit and explicit measures of fear of weight gain were non-significant.

At first glance, the lack of group differences between participants with FP-AN and HC with regard to the relative importance of appearance seems counterintuitive, as patients with AN often seem to sacrifice important aspects of their lives in order to maintain their low weight (Serpell et al., 1999; Roux et al., 2016). Also, when evaluating self-worth, the overpowering predominance of shape and weight over other important aspects in life in samples with ED becomes evident (Fairburn et al., 2008). In contrast, in this sample, the relative importance of “Appearance/Shape and Weight” was low, being in fourth and fifth place. As participants in this study were asked to choose the life profiles they would wish most for, rather than the profile that most closely resembled their current life, our results may be evidence of the disconnect between what patients with AN wish for and what they achieve in their lives, rather than an indication of their current attitude toward weight gain. Furthermore, as the majority of our patients with AN endorsed in past psychotherapy, they might have already worked on this topic during their sessions. Exploring and understanding these results in greater detail, it is important to examine the preferences for the attributes within this factor. As expected, participants with FP-AN showed a significantly lower preference for weight gain versus weight maintenance compared to HC. The difference between the part-worth utilities for both attributes is much higher for HC than for participants with FP-AN, signifying that the preference for weight gain in HC is more pronounced than the opposing preference in participants with FP-AN. This suggests that HC may experience less ambivalence regarding the choice between weight maintenance and gain and may actively choose weight gain in order to secure positive rewards in other areas of their lives compared to individuals with FP-AN.

Evaluating the relative importance of the other factors of the life profiles, our results revealed no significant differences in the relative importance placed on the various factors between FP-AN and HC. This supports a study on explicit life goals in patients with AN and BN compared to HC (Hötzel et al., 2012) showing that individuals with AN and BN generally pursued the same goals in life as HC, but exhibited deficits in goal realization.

A preliminary exploratory subgroup analysis on participants with NFP-AN and HC also revealed no significant difference in the relative importance of appearance between the two groups. Yet, looking at the part-worth utilities, it is of note that NFP-AN participants’ preference for weight maintenance vs. weight gain became apparent, which was even more pronounced than those of participants with FP-AN, still tentatively suggesting that contrary to their self-report, they may experience fear of weight gain. These results are in line with the findings on adolescents and young women with NFP-AN (Izquierdo et al., 2019) and could reflect an explicit denial of fear of weight gain while facing an unconscious fear of weight gain at the same time. It is also of note that participants with NFP-AN put a significant higher relative importance on success in job or school while they showed a significantly lower relative importance on the factor interpersonal/belonging than HC. High importance of being successful could be related to the high level of perfectionism found in samples with AN (Hartmann et al., 2014) which could be even higher in NFP-AN. It may also be that being successful in job or school is more compatible with a very low weight than maintaining a good relationship with friends and family. Further support for this possible explanation is the finding that, also in line with prior research (Santonastaso et al., 2009), the BMI of our individuals with NFP-AN was even lower than in FP-AN. Yet, against this hypothesis, participants with FP-AN showed a significant lower preference for being successful in job or school than HC. Taking all together, these preliminary results may be interpreted in a way that NFP-AN seem to be less ambiguous in their life choices, including their weight, and therefore differ more in their profile from HC than FP-AN. Yet, the findings regarding the comparison of NFP-AN and HC have to be interpreted with caution due to the small sample size of participants with NFP-AN.

For a dimensional analysis of implicit fear of weight gain in AN, we were also interested in the correlations between importance and preference score and questionnaire-based measures of fear of weight gain and behavioral manifestations of a body-image disturbance. The direction of the correlations between the questionnaire-based measures and the part-worth utility of the attribute within “Appearance/Shape and Weight” were as expected—negative for “Gained 3 kg in the last 2 months and don’t fit into your favorite jeans.” Yet, contrary to our expectations, all correlations were non-significant and small, again suggesting only a tenuous connection between what was measured with the help of the questionnaires and what was assessed through the CBC analysis. Several reasons for the discrepancy of implicit and explicit measures have been discussed in literature (e.g., Hofmann et al., 2005; Roefs et al., 2011), e.g., motivational biases in self-reports, independence of the underlying constructs, and lack of introspection. Both the characteristic of the CBC instruction and the idea of a minimizing response style in NFP-AN (Izquierdo et al., 2019), these factors could play a role for these results left open to discuss.

This piece of research was subject to certain limitations. The sample size for the group of participants with NFP-AN was very small, resulting in low statistical power which may mean small statistical differences were not detected and results for participants with NFP-AN should be interpreted with caution. Further research with larger sample sizes should be carried out on NFP-AN to allow for more definite and generalizable conclusions. Although the NFP phenotype seems to be stable during treatment (Dalle Grave et al., 2008; Carter and Bewell-Weiss, 2011) and over 12-month follow-up (Wildes et al., 2013), we cannot rule out that previous treatment changed the EDI-2 Drive for Thinness score used to categorize the two AN subgroups. Furthermore, the HC group mainly consisted of students between the ages of 19 and 28, calling into question the representativeness of this group for the general population. It also needs to be considered whether the instruction given during the CBC analysis, to choose the life profile most wished for, was appropriate for assessing the current feelings of the participants toward weight gain. The wording of the task was originally based on the idea that the core concept, i.e., fear of weight gain, is rather future-oriented in nature (Murray et al., 2016). As touched on above, the results may be more indicative of the future goals and wishes of the participants rather than their present state of mind. Asking participants to, for example, choose the life profile they most identified with might have been a more suitable directive considering the research question. Looking at the strengths of this study, this was the first study implicitly assessing fear of weight gain in adult participants with NFP- and FP-AN who had been thoroughly diagnosed using a structured clinical interview. We employed CA (a reliable and popular method in commercial settings) as a novel method in this clinical context.

Conclusively, it can be said that the results of this study indicate that we did succeed in measuring fear of weight to some extent. The preferences for the attributes within the factor “Appearance/Shape and Weight” revealed the marked preference for weight maintenance of participants with AN and for weight gain in HC which is in line with our expectations and thus confers face validity. Participants with NFP-AN showed a strong preference for weight maintenance indicating that, in line with Izquierdo et al. (2019), they may experience fear of weight gain though explicitly denying it. However, the real and sole influence of fear of weight gain on the answering pattern is hard to distinguish in this paradigm, as the results for all factors and attributes are relative to and impact each other. It thus can be concluded that there is some utility of CA as a diagnostic tool for measuring fear of weight gain and it might be of great value in clinical and especially therapeutic settings as a means for therapists and patients to gain insight into the specific goals of patients and to identify potential conflicts between these goals.

## Data Availability Statement

The datasets generated for this study are available on request to the corresponding author.

## Ethics Statement

The study protocol was approved by the Ethics Committee of Osnabrück University. Written informed consent to participate in this study was provided by the participants and (if minor) her/his legal guardian/next of kin.

## Author Contributions

JT and AH planned the study. LR and AH carried out the assessment. SV supervised it. JK analyzed the data and wrote the manuscript. All authors critically edited the manuscript.

## Conflict of Interest

The authors declare that the research was conducted in the absence of any commercial or financial relationships that could be construed as a potential conflict of interest.
